# Losartan improves the therapeutic effect of metronomic cyclophosphamide in triple negative mammary cancer models

**DOI:** 10.18632/oncotarget.27694

**Published:** 2020-08-11

**Authors:** Leandro E. Mainetti, María José Rico, Cintia Daniela Kaufman, Monica Carolina Grillo, Julian Guercetti, María Virginia Baglioni, Antonela Del Giúdice, Maria Celeste Capitani, Matias Fusini, Viviana Rosa Rozados, O. Graciela Scharovsky

**Affiliations:** ^1^Instituto de Genética Experimental, Facultad de Ciencias Médicas, Universidad Nacional de Rosario, Rosario, Argentina; ^2^Consejo Nacional de Investigaciones Científicas y Técnicas, Buenos Aires, Argentina; ^3^Metronomics Global Health Initiative, Marseille, France; ^*^These authors contributed equally and are co-first authors; ^#^These authors contributed equally and are co-senior authors

**Keywords:** mammary cancer, metronomic chemotherapy, drug repurposing, cyclophosphamide, losartan

## Abstract

Metronomic chemotherapy refers to the minimum biologically effective doses of a chemotherapy agent given as a continuous regimen without extended rest periods. Drug repurposing is defined as the use of an already known drug for a new medical indication, different from the original one. In oncology the combination of these two therapeutic approaches is called “Metronomics”.

The aim of this work is to evaluate the therapeutic effect of cyclophosphamide in a metronomic schedule in combination with the repurposed drug losartan in two genetically different mice models of triple negative breast cancer.

Our findings showed that adding losartan to metronomic cyclophosphamide significantly improved the therapeutic outcome. In both models the combined treatment increased the mice’s survival without sings of toxicity. Moreover, we elucidated some of the mechanisms of action involved, which include a decrease of intratumor hypoxia, stimulation of the immune response and remodeling of the tumor microenvironment.

The remarkable therapeutic effect, the lack of toxicity, the low cost of the drugs and its oral administration, strongly suggest its translation to the clinical setting in the near future.

## INTRODUCTION

Cancer is a disease caused by the accumulation of genetic and epigenetic changes. According to global cancer statistics, about 2.1 million female breast cancer were to be diagnosed in 2018, thus accounting for almost 1 in 4 cancer cases among women [1]. Standard clinical protocols for cancer chemotherapy typically employ the maximum drug dose that can be tolerated by the patient (MTD). These regimens lead, in turn, to the need for drug-free periods between treatment cycles to allow normal tissue recovery from the cytotoxic attack; they are ideally designed to maximize tumor cell killing without lethal damage to the patient. Nevertheless, as a counterpart of its effectiveness, its high toxicity has detrimental effects on the patients’ quality of life and, also, the treatment can lead to the development of therapeutic resistance [2]. In contrast to MTD regimens, metronomic chemotherapy (MCT) is characterized by the chronic, equally spaced administration of (generally) low doses of chemotherapeutic drugs, without extended rest periods [3–5]. The novelty of this treatment modality, known as anti-angiogenic therapy, lies in its main cell target, the tumor endothelial cell [6]. Also, another mechanism of action of MCT include activation of anti-tumor immunity [7].

Drug repurposing is a creative and resourceful approach to increase the number of therapies by exploiting available and already approved drugs. In oncology, drug repurposing is gathering momentum, mainly because revolutionary advances in pharmacology and genomics have demonstrated that many old drugs, designed for other indications, have antitumor activity [8]. Drug repurposing is frequently used with MCT, a combination of therapeutic approaches that has been defined as “Metronomics” [9].

Cyclophosphamide (Cy), one of the most frequently used alkylating agents, has shown antitumor activity when administered with a metronomic schedule [10–12].

Losartan (Los), is an angiotensin II receptor type 1 antagonist, approved to control hypertension in patients [13] and, it is also an antifibrotic agent shown to reduce the incidence of cardiac and renal fibrosis [14, 15]. As a repurposed drug for cancer treatment, Los reduces stromal collagen, improving drug and oxygen delivery to tumors, thereby potentiating chemotherapy and reducing hypoxia in breast and pancreatic cancer models [16].

We report here that the combined metronomic treatment of Cy plus Los, used in two triple negative mammary adenocarcinoma murine models, inhibited tumor growth, increased survival and was devoid of general toxic effects, showing that Los can increase the therapeutic effectiveness of MCT with Cy.

## RESULTS

### Antitumor effect

**Figure 1:**
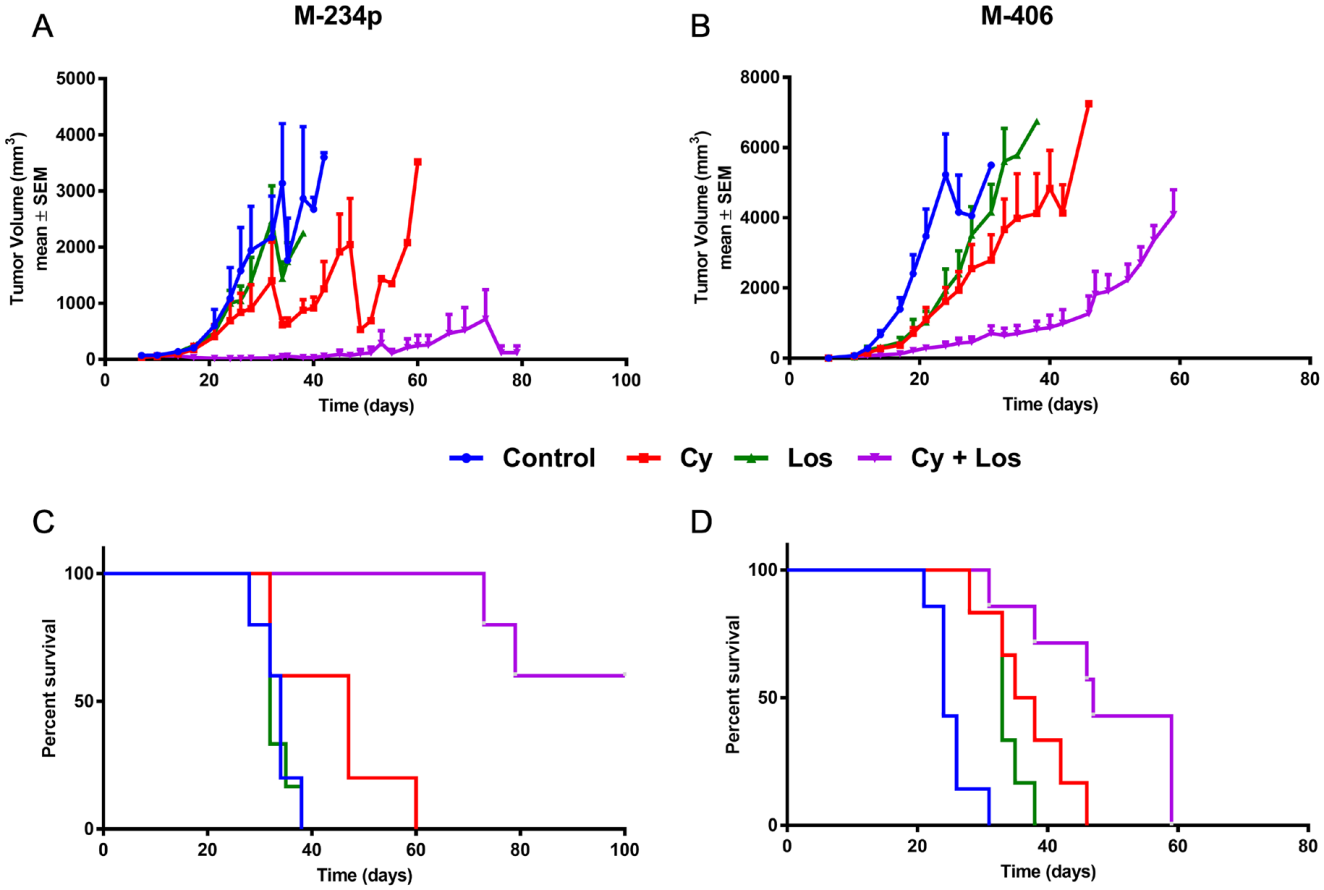
Tumor growth and overall survival. Tumor growth assessment: data for each time-point in mm^3^ are mean ± SEM. (**A**) M-234p, *N* = 6/group; Day 21: Control (600.96 ± 93) vs Cy+Los (20.96 ± 9.01) (*P* < 0.05); (**B**) M-406, *N* = 6–7/group; Day 17: Control (1397.00 ± 328.32) vs Cy (372.00 ± 55.25) (*P* < 0.01), vs Los (451.07 ± 143.94) (*P* < 0.05), vs Cy+Los (123.43 ± 45.71) (*P* < 0.001). Kruskal-Wallis multiple comparison test and Dunn’s post-test. Overall survival (Kaplan-Meier), Median Survival (MS): (**C**) M-234p, *N* = 5–6/group; Control (MS: 34 days); Cy (MS: 47 days); Los (MS: 32 days); Cy+Los (MS: undefined, Day 32: 60% [3/5] complete tumor regressions). Cy+Los vs Control, vs Los, vs Cy (*P* < 0.01); (**D**) M-406, *N* = 6–7/group); Control (MS: 24 days); Cy (MS: 36.5 days); Los (MS: 33 days); Cy+Los (MS: 47 days). Control vs Cy (*P* < 0.01), vs Los (*P* < 0.01), vs Cy+Los (*P* < 0.001); Cy+Los vs Cy (*P* < 0.05), vs Los (*P* < 0,001). Log-rank Test.

The effect on tumor growth of MCT with low doses Cy plus Los was studied by implanting orthotopically in the mammary fat pad, M-234p or M-406 mammary adenocarcinomas in BALB/c or CBi mice, respectively. As can be seen in Figure 1A, the treatment with Cy+Los significantly inhibited the growth of M-234p tumors, compared to non-treated Control and Los groups, from day 21 onwards (*P* < 0.05). A similar effect was observed in the M-406 tumor model (Figure 1B). The mean volume of M-406 tumors in Control group was higher, on day 17, than in that of Cy (*P* < 0.01), Los (*P* < 0.05) and Cy+Los (*P* < 0.001) groups.

### Survival

As a consequence of the anti-tumor effect, there was an increase in survival. M-234p bearing mice that received the combined treatment Cy+Los showed a significantly higher survival than that of Control group (Median Survival [MS]: 34 days; *P* < 0.01), Cy (MS: 47 days; *P* < 0.01), and Los (MS: 32 days; *P* < 0.01) (Figure 1C), while Control, Cy and Los groups did not differ from each other. Importantly, by day 60, all the mice in Control, Cy and Los groups had already been sacrificed, because of their tumor burden, but none of the mice in the Cy+Los group. In the combined treatment group, two animals were sacrificed on days 73 and 79, respectively, while the remaining three animals (3/5; 60%) showed complete tumor regression (MS: undefined). The treatment of these mice was discontinued on day 80. Interestingly, after 20 more days without treatment, until the end of the experiment (Day 100), no recurrences were detected.

In the M-406 tumor model, when compared to Control (MS: 24) group, the different treatments significantly increased survival, Cy (MS: 36.5 days; *P* < 0.01), Los (MS: 33 days; *P* < 0.01), and Cy+Los (MS: 47 days; *P* < 0.001). Also, Cy+Los group showed a significantly higher survival than Cy (*P* < 0.05) and Los (*P* < 0,001). In spite of the fact that no tumor regressions were achieved in this tumor model, the median survival time in Cy+Los group almost duplicated that of the Control group (24 vs 47 days) (Figure 1D).

### Toxicity

When monitoring several signs related to toxicity in both tumor models, treated animals showed normal traits in food intake, response to stimuli, motor activity, breathing and fur quality. Also, the treatments did not cause weight loss (Supplementary Figure 1).

### Mechanisms of action

#### Proliferation

**Figure 2:**
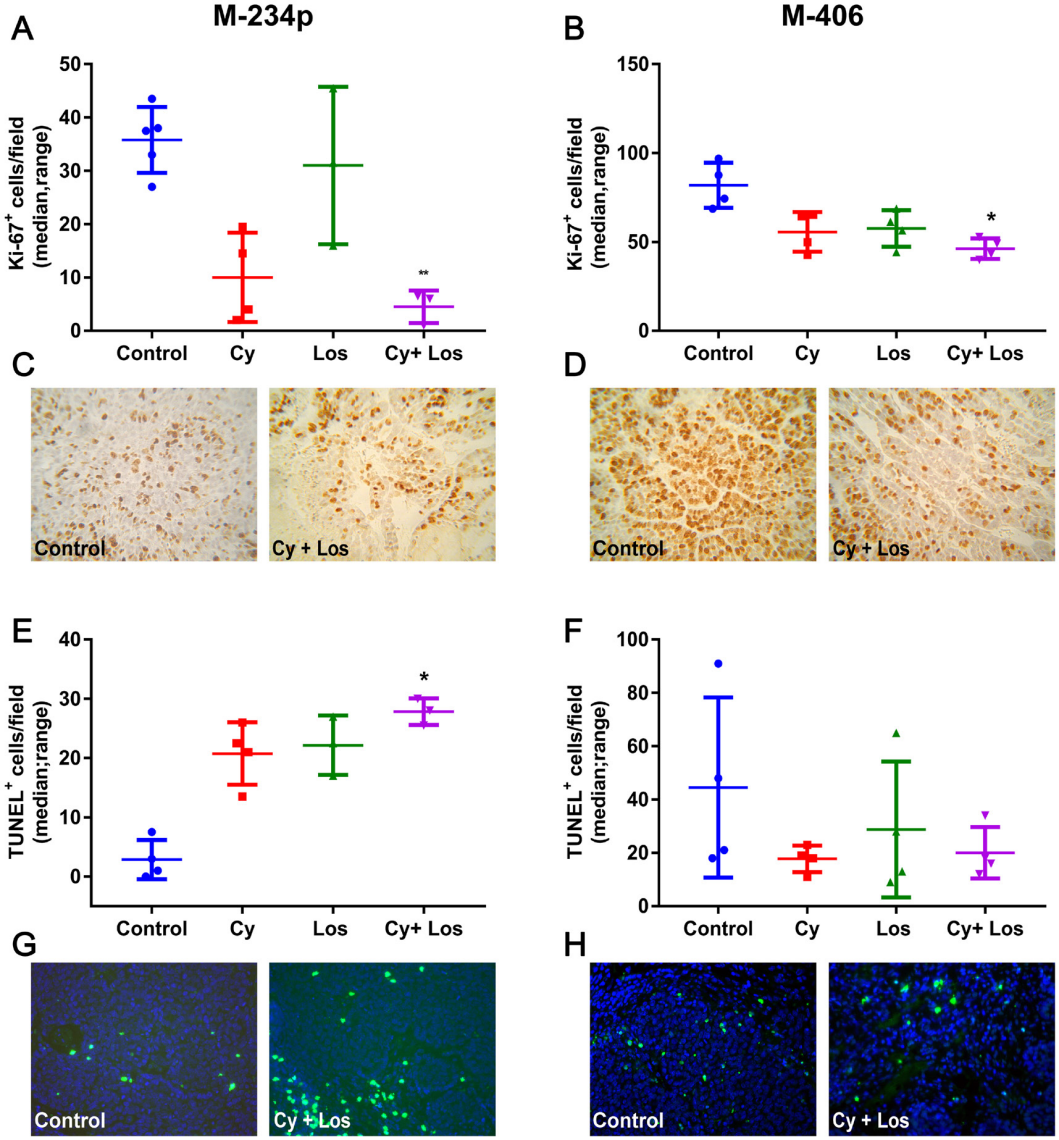
Ki67 expression and apoptosis quantification. Proliferation: Ki67^+^ cells/field (median, range). (**A**) M-234p Control vs Cy+Los (*P* < 0.01); (**B**) M-406 Control vs Cy+Los (*P* < 0.05; (**C**) M-234p and (**D**) M-406, representative images of Control and Cy+Los treated tumors, 1000× magnification. Apoptosis: TUNEL^+^ cells/field (median, range). (**E**) M-234p Control vs Cy+Los (*P* < 0.05): (**F**) M-406 N. S; Kruskal-Wallis multiple comparison test and Dunn’s post-test; (**G**) M-234p and (**H**) M-406 representative images of Control and Cy+Los treated tumors, 1000× magnification.

In the M-234p and M-406 tumor models, the group treated with Cy+Los showed significantly lower Ki67 tumor expression when compared to Control group (*P* < 0.01 and *P* < 0.05, respectively) (Figure 2A–2D).

#### Apoptosis

In the M-234p tumor model, the number of TUNEL^+^ cells in Cy+Los group was higher than in that of Control group (*P* < 0.05), (Figure 2E and 2G). On the other hand, no significant differences were found among groups in the M-406 model (Figure 2F and 2H).

#### Angiogenesis

**Figure 3:**
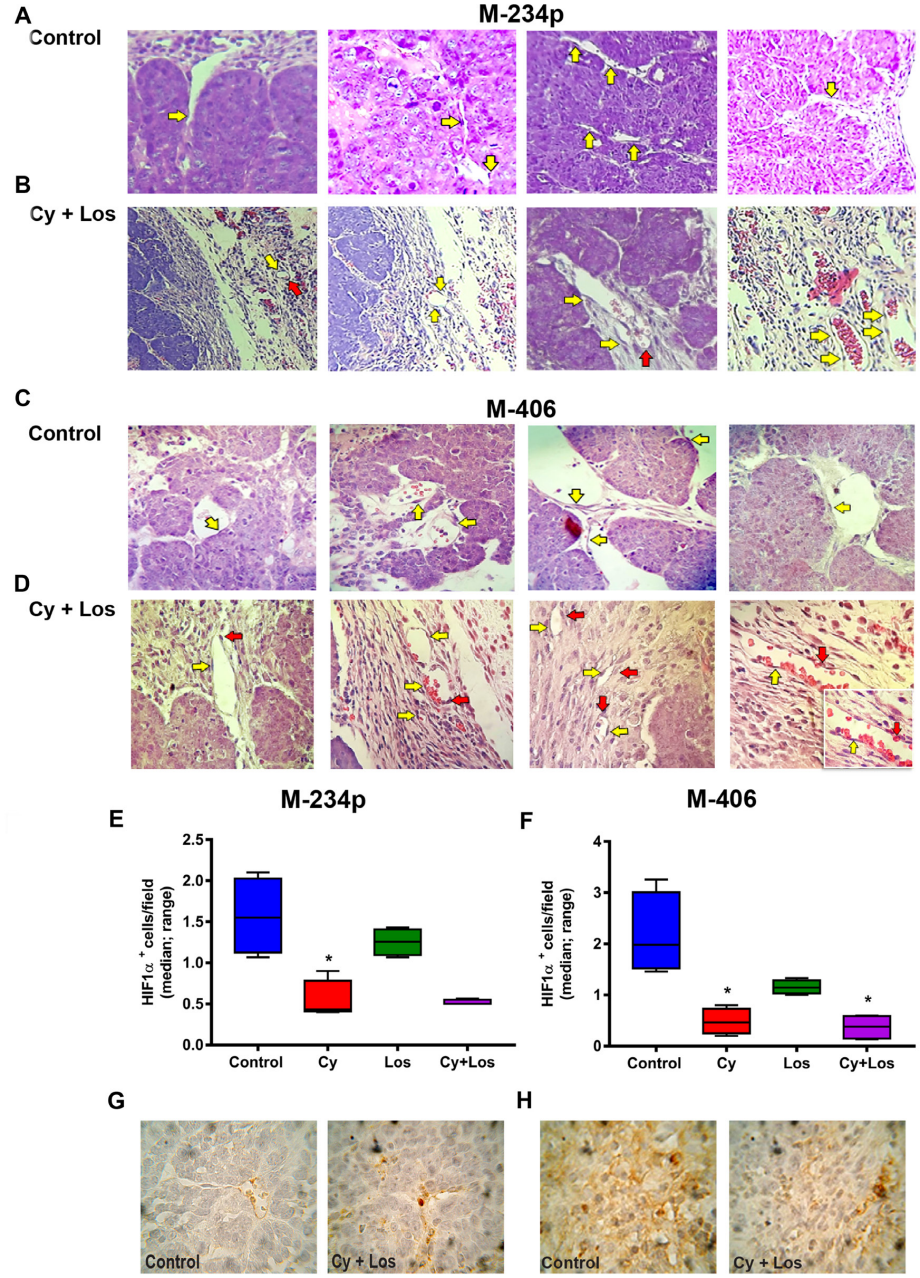
Vascular normalization. Hematoxylin and eosin (H&E) representative tumor sections from M-234p and M-406, 400×. In both models the behavior was similar. Control group: (**A**) M-234p and (**C**) M-406: capillaries with small endothelial cells with barely stained nuclei and intercellular gaps (yellow arrow), lack of pericytes or cells with structure and staining compatible with pericytes. Cy+Los group: (**B**) M-234p and (**D**) M-406: intra- and peritumoral capillaries with structure and morphology similar to normal tissues. Endothelial cells with defined nuclei provide a continuous uninterrupted lining (yellow arrow), and well defined basal membrane covered with pericytes (red arrow). M-406 magnified section (1000×): vessel with normal vascular morphology. HIF1α expression: HIF1α^+^cells/field (median, range). (**E**) Control vs Cy (*P* < 0.05), (**F**) Control vs Cy (*P* < 0.05), vs Cy+Los (*P* < 0.05), (**G**) and **H**), representative images of Control and Cy+Los treated tumors, 100× magnification. Kruskal-Wallis multiple comparison test and Dunn’s post-test.

The morphology of tumor blood vessels was analyzed in samples stained with H&E by a trained pathologist. The results were similar for both tumor models. The capillaries in Control group samples showed a thin and interrupted connective tissue sheet. The inner layer is characterized by small endothelial cells with flat nuclei and intercellular gaps forming a discontinuous vessel. It is important to notice the lack of pericytes (or cells that by structure and staining are compatible with pericytes) surrounding the capillary. On the other hand, samples from the group treated with Cy+Los showed intra- and peritumoral capillaries with structure and morphology similar to capillaries that irrigate normal tissues. Endothelial cells provide a continuous uninterrupted lining, with a complete and well defined basal membrane covered with pericytes. Samples from tumors belonging to Cy or Los groups showed vessels and capillaries with a structure and morphology similar to Control group (Figure 3A–3D). Furthermore, the effect of the treatment on intratumor hypoxia was analyzed by immunohistochemistry with HIF1-α expression. The treatment with Cy, in both tumor models, and with Cy+Los in M-406 tumors, decreased significantly the number of HIF1-α positive cells per field compared to Control group (*P* < 0.05) (Figure 3E–3H).

#### Immune cells populations

**Figure 4:**
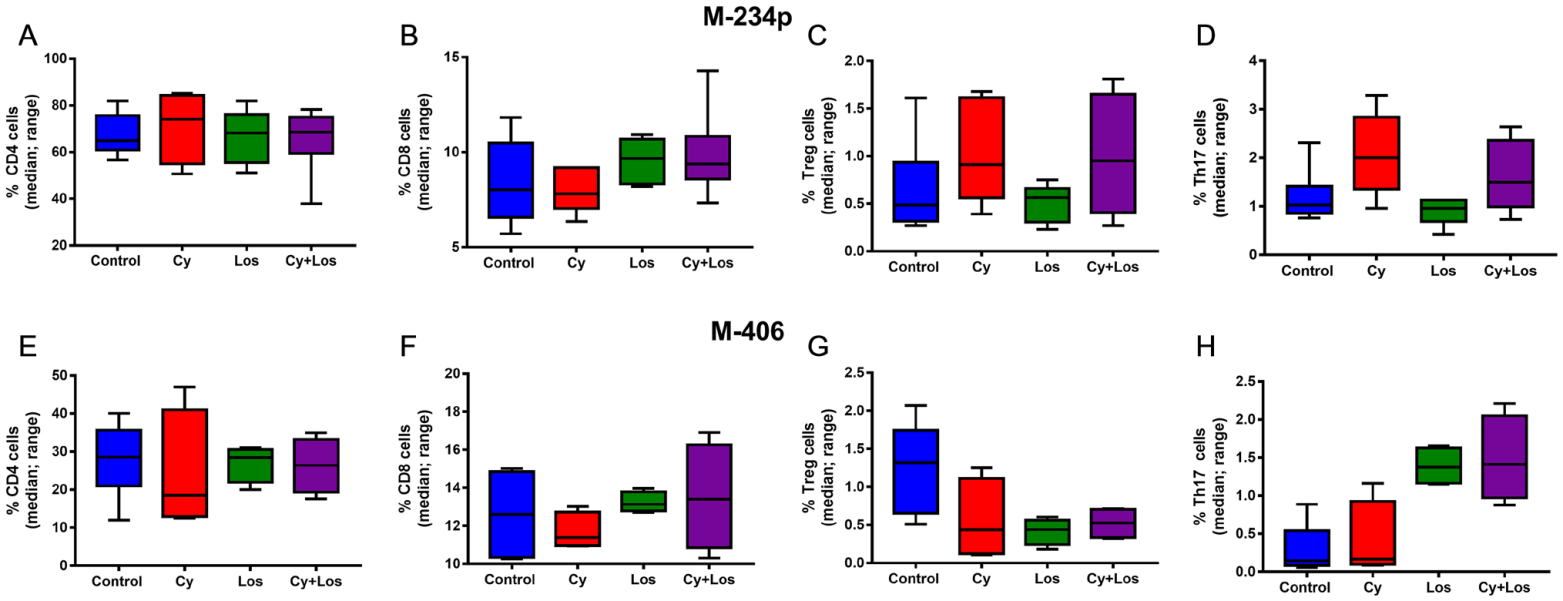
Quantification of circulating T cells by flow cytometry: % of positive cells (median, range). M-234p: (**A**) CD4 cells, N. S. (**B**) CD8 cells, N. S. (**C**) Treg cells, N. S. (**D**) Th17 cells, N. S. M-406: (**E**) CD4 cells, N. S. (**F**) CD8 cells, N. S. (**G**) Treg cells, (*P* = 0.064). (**H**) Th17 cells: Control vs Cy+Los, (*P* = 0.0580). Kruskal-Wallis multiple comparison test and Dunn’s post-test.

The percentage of circulating CD4^+^ and CD8^+^ cells did not show, on day 30, significant differences among groups in any of the two models (Figure 4A, 4B, 4E and 4F). Also, in the M-234p model there were no differences among groups in circulating Treg (Foxp3^+^) and Th17 cell populations (Figure 4C and 4D); on the contrary, in the M-406 model the number of Treg cells in Los and Cy+Los groups were lower (*P* = 0.064), and the number of Th17 cells were higher in the same groups (*P* < 0.01), when compared to Control group (Figure 4G and 4H).

**Figure 5:**
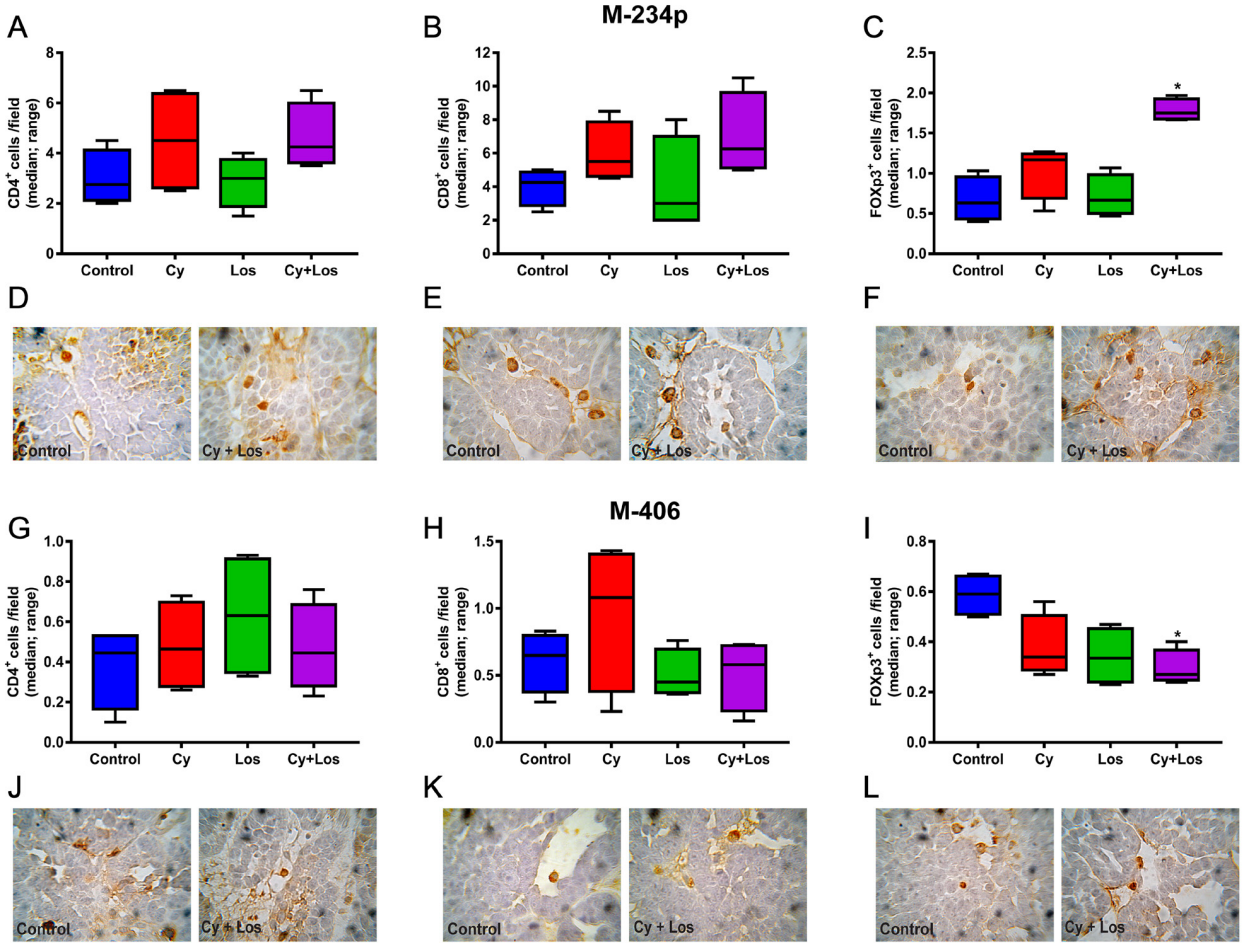
Quantification of tumor infiltrating lymphocytes by IHC. Lymphocytes/field (median, range). M-234p: (**A**) CD4^+^ cells, N. S. (**B**) CD8^+^ cells, N. S. (**C**) Foxp3^+^ cells: Control vs Los, *P* < 0.05, vs Cy+Los, (*P* < 0.05); (**D**–**F**) representative images of Control and Cy+Los treated tumors, 100× magnification; M-406: (**G**) CD4^+^ cells, N. S. (**H**) CD8^+^ cells, N. S. (**I**) Foxp3^+^ cells: Control vs Cy+Los, (*P* < 0.05); (**J**–**L**) representative images of Control and Cy+Los treated tumors, 100× magnification. Kruskal-Wallis multiple comparison test and Dunn’s post-test.

**Figure 6:**
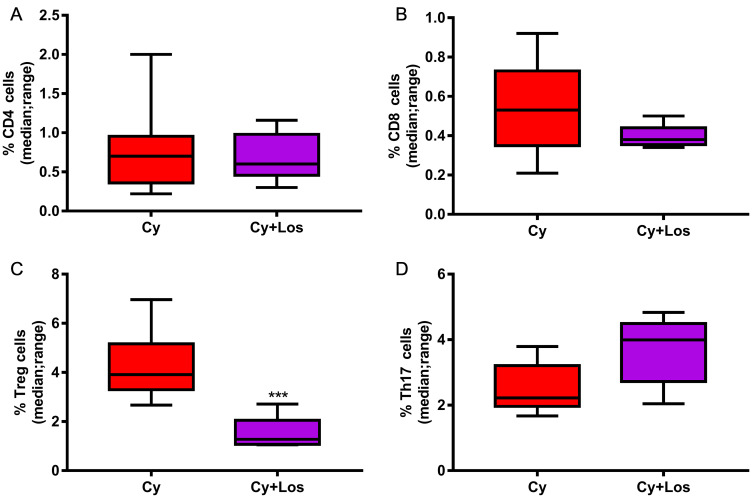
Quantification of tumor infiltrating lymphocytes by flow cytometry: M-234p, day 42, Cy vs Cy+Los: (**A**) CD4 cells, N. S. (**B**) CD8 cells, N. S. (**C**) Treg cells, (*P* < 0.001). (**D**) Th17 cells, (*P* < 0.05). Kruskal-Wallis multiple comparison test and Dunn’s post -test.

When quantifying the tumor infiltrating CD4^+^ and CD8^+^ cells, no significant differences among groups were found, on day 30, in none of the two models (Figure 5A, 5B, 5D, 5E, 5G, 5H, 5J and 5K). Besides, the analysis of the tumor infiltrating Treg cells in samples taken on day 30, showed the opposite behavior in each tumor model. Samples of M-234p tumors from animals treated with Cy+Los had a significantly higher number of Foxp3^+^ cells compared to that of Control group (*P* < 0.05) (Figure 5C and 5F). On the other hand, in the M-406 tumor samples, a significant decrease in the number of Treg cells in Cy+Los group (*P* < 0.05) (Figure 5I and 5L) was found. Moreover, M-234p tumor samples were taken on day 42, a time point when all the mice in Control and Los groups were euthanized. Tumor infiltrating lymphocytes populations were analyzed by flow cytometry. Interestingly, at this time, the number of Treg cells was significantly lower and Th17 cells significantly higher in Cy+Los group compared to those of Cy group (*P* < 0.001 and *P* < 0.05, respectively) (Figure 6C and 6D). As in day 30 quantifications, no differences were found for the CD4^+^ and CD8^+^ cells (Figure 6A and 6B).

#### Cancer Associated Fibroblasts (CAF)

**Figure 7:**
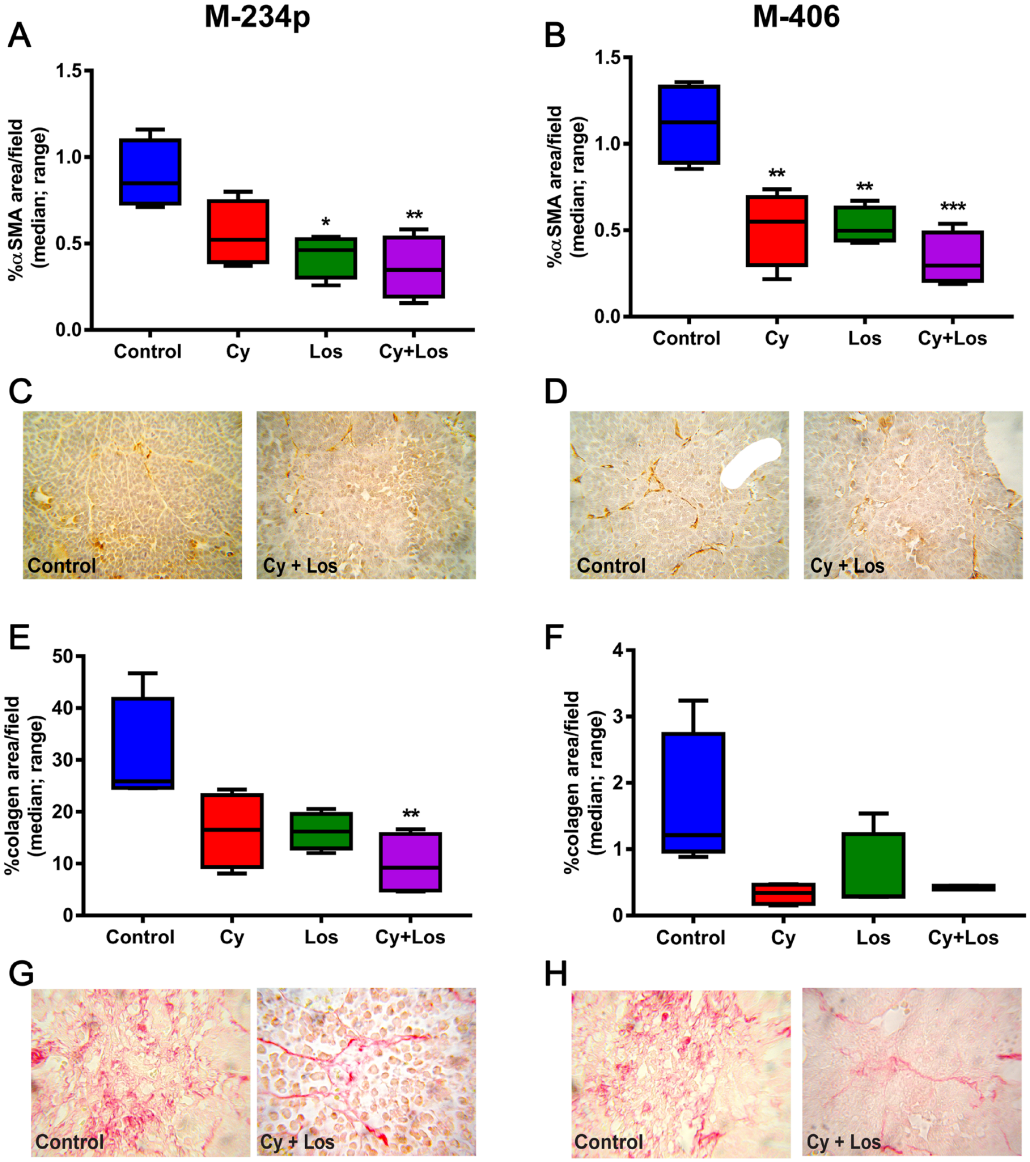
αSMA expression and collagen area. *αSMA*: % of *αSMA*^+^ area/field (median, range). (**A**) M-234p Control vs Los, (*P* < 0.05), vs Cy+Los, (*P* < 0.01). (**B**) M-406 Control vs Cy, (*P* < 0.01), vs Los (*P* < 0.01), vs Cy+Los (*P* < 0.001). (**C**) M-234p and (**D**) M-406 representative images of Control and Cy+Los treated tumors, 100× magnification. Kruskal-Wallis multiple comparison test and Dunn’s post-test. *Collagen*: % of collagen area/field (median, range). (**E**) M-234p Control vs Cy+Los (*P* < 0.01). (**F**) M-406 N. S. (**G**) M-234p and (**H**) M-406 representative images of Control and Cy+Los treated tumors, 100× magnification.

The number and activity of CAF were evaluated by IHC through the αSMA expression and the Picro Sirius Red staining for collagen quantification. The % of αSMA^+^ area was significantly lower in the Cy+Los group when compared to Control animals in both tumor models (*P* < 0.01) (Figure 7A–7D). The area occupied by collagen in the tumor samples of the three treated groups in both models was lower than that of Control group, showing a significant difference between Cy+Los and Control groups in the M-234p model (*P* < 0.01) (Figure 7E–7H).

## DISCUSSION

Metronomic chemotherapy has come a long way since the first publications in 2000 [3, 5]. This therapeutic approach has demonstrated its efficacy for the treatment of different types of cancer [17–20]. Cyclophosphamide is among the first cytotoxic drugs used in a metronomic schedule [21, 22]. In our own experience, we have administered metronomic Cy, alone or in combination with other drugs, in several preclinical models and in a clinical trial [10, 11, 23–25]. Losartan has been used lately as repurposed drug in cancer treatment [26, 27]. Although its therapeutic effect as a single drug is poor, its combination with other antitumor agents highly enhances its efficacy. Such effect could be due, in part, to a decrease in the extracellular matrix collagen expression which, in turn, would improve drug delivery into the tumor [28, 29].

We found that the combined treatment with Cy+Los, in both triple negative mammary tumor models, inhibited tumor growth and, interestingly, was more efficient than the treatments with each drug alone, resulting in a very significant increase in the overall survival. Importantly, in the M-234p tumor model, the combined treatment led to a 60% of tumor regressions, which lasted, without recurrences, even 20 days after discontinuation of therapy. On the other hand, no regressions were observed in M-406 tumor model. Nevertheless, the encouraging result was that the combined treatment duplicated the median survival time of M-406 tumor bearers.

The observation of different behaviors in the tumor models is interesting, because, as it is the case in human cancers, the different genetic backgrounds in hosts and, therefore, in their tumors, lead to different therapeutic outcomes. In the development of a malignant tumor and of the derived metastasis are involved a high number of both, genetic and epigenetic events. Hence, those characteristics will shape the response to treatments of different lines of mice.

One of the advantages of the therapeutic approach called *Metronomics* is that it has low toxicity [30]. In fact, we found that the treatment did not cause weight loss, nor did it cause any alterations in the markers of morbidity/toxicity monitored, in none of the two tumor models.

Nowadays, metronomic chemotherapy is defined as a multitarget therapy [31], a fact that suggests the evaluation of different mechanisms of action that may be involved in the therapeutic effect.

The combined treatment significantly diminished the expression of the proliferation marker Ki67 in both tumor models and, simultaneously, increased the number of apoptotic cells in M-234p tumor, while there were no modifications in M-406 tumor. The induction of apoptosis in M-234p tumor cells would be responsible, at least in part, for the higher efficacy of the treatment in this tumor model.

The first and most studied mechanism of action of metronomic chemotherapy was its effect on angiogenesis. The antiangiogenic effect of Cy has been widely validated [5, 17, 32]. Moreover, other authors have already demonstrated that Los can cause inhibition of angiogenesis [28, 33]. Recently, several studies have demonstrated that the main effect of the antiangiogenic activity of metronomic chemotherapy is the vascular normalization [34]. Metronomic Cy was demonstrated to improve drug uptake and tumor oxygenation in a preclinical model [35]. Besides, Los diminishes the solid stress that compresses tumor blood vessels, achieving a similar result to vascular normalization through microenvironment normalization; this effect is mediated by the reduction of collagen production which results in decompression of tumor capillaries [16]. A qualitative analysis of the tumor samples from the different groups has indicated that only the combined treatment produced a morphological and structural switch that resembles a vasculature found in normal mammary tissue, while neither Cy nor Los produced, individually, any change compatible with vascular normalization. On the other hand, in both tumor models, we found a significant and similar decrease of HIF1-α in tumor samples from animals treated with Cy alone or with Cy+Los. This outcome indicates, at least from the HIF1-α perspective, no participation of Los on angiogenesis, while it confirms the antiangiogenic effect of Cy previously observed [11]. These results indicate that the treatment with Cy plus Los may produce vascular normalization with vessels that have similar morphology to that of normal mammary tissue, increasing tumor oxygenation, reducing hypoxia and, as a consequence, improving the intratumor distribution and delivery of drugs, hence leading to a better therapeutic outcome.

As demonstrated by several groups, included our own, the administration of Cy in a metronomic fashion can improve the antitumor immune response [24, 36]. Metronomic administration of cyclophosphamide, have been proved to stimulate the immune response against the tumor, mainly by reducing regulatory T cells and increasing Th17 cells populations [37]. On the other hand, there is less evidence showing the effect of Los on the immune response. It was observed, *in vitro*, that Los decreased the secretion of TGF-β by mononuclear cells [38]. Gabriele et al., suggested that Los may differently control the balance of inflammatory cytokines such as IL-6 and IL-8, in human periodontal fibroblasts [39]. Also, Coulson associated the inhibition of progression with reduction of mammary tumor cell proliferation and of pSTAT3, TNFα and IL-6 in Losartan-treated group compared to control group [40]. Based on those previous works, we wanted to know the behavior of different T cells populations when administering the novel combination of cyclophosphamide and losartan in mice bearing mammary tumors. In the present study we analyzed different populations of circulating and tumor infiltrating T lymphocytes to elucidate their role in the therapeutic effect. The percentage of CD4, CD8, Tregs and Th17 circulating cells showed no changes induced by the treatments in the M-234p model. On the contrary, in the M-406 model, there was a marginally significant decrease in Tregs and a significant increase in Th17 cells in the combined treated group. Both modifications may contribute to the therapeutic effect obtained.

The evaluation of the tumor infiltrating regulatory T cells in tumor samples taken on day 30, revealed opposite results in the two models. The number of Treg cells in M-234p samples of Cy+Los group, was significantly higher than those in the other groups, while in the M-406 samples of the combined treated group, the percentage of these cells was significantly lower, when compared to Control tumors. The M-406 results were in agreement with those obtained for circulating Tregs. On the other hand, those obtained in M-234p tumors, were the opposite of the expected ones. The idea that maybe the kinetics of Tregs participation could be different in this particular tumor model, led us to quantify this cell population in samples taken on the 42nd day of tumor evolution. Indeed, that was the case because, at this time point, in which the tumor volume of the Cy+Los group was significantly lower than that of the Cy treated group, Tregs cells had also decreased with respect to Cy group. Moreover, a significant increase of Th17 cells in the combined treated group was also observed. Thus, the diminution of suppressor Treg and the increase of Th17 cells populations may play an antitumor role at this stage of tumor treatment.

Therefore, it is clear that while the final results showed the same tendency in both tumor models, the kinetics of participation of immune cells populations was quite different. Such result is not surprising, considering that each tumor may well differ, not only in their immunogenicity but also in the crucial aspect of the interaction between the tumor and its microenvironment. Other cell populations involved in the anti-tumor immune response like macrophages or myeloid derived suppressor cells will be analyzed in future experiments.

The effect of Los on the tumor micro environment has already been reported [28, 41]. According to these authors, the main target of Los are the cancer associated fibroblasts (CAF), causing a reduction in the amount of collagen I, which thereby allowed a better distribution of any cytotoxic agent. We used the expression of αSMA and the amount of collagen as markers of CAF. In the present work, the therapeutic effect of Cy was significantly improved by the combination with Los in both tumor models. The immunohistochemistry analysis of tumor samples showed that the combined treatment significantly diminished the expression of αSMA and the collagen deposition, indicating an inhibitory effect on the CAF population. The therapeutic results obtained with the combined treatment, could be explained, on the one hand, by the inactivation produced by Los on CAF and, on the other hand, by a putative mild cytotoxic effect that Cy could have on the same type of cells when, in spite of the low doses administered, they can reach more easily the tumor cell, due to the decrease in collagen in the tumor microenvironment.

To the best of our knowledge, this paper describes for the first time, the metronomic treatment of triple negative breast cancers with a combination of cyclophosphamide and losartan. The antitumor effect of the drugs combination caused not only a significant diminution in tumor growth and a higher overall survival, but also the achievement of complete tumor regressions in one of the tumor models. Importantly, the treatment was devoid of general toxicity, without showing weight loss, in spite of the long period of daily medication. We also gained insight into some of the mechanisms responsible for the therapeutic effect obtained.

The therapeutic achievements obtained with Cy+Los treatment that were, by far, better than those obtained by us, utilizing different drug combinations [11, 12, 42], together with other advantages like low cost, oral administration and lack of toxicity, leading to a better quality of life, strongly suggest its translation to the clinical setting in the near future.

## MATERIALS AND METHODS

### Animals

Six week-old inbred CBi [43] and BALB/c female mice were obtained from the Institute of Experimental Genetic breading facilities. Animals were fed with commercial chow and water *ad libitum* and were maintained in a 12-h light/dark cycle. All the experiments were developed during the first half of the light cycle. The animals were treated in accordance to the Canadian Council on Animal Care guidelines.

### Drugs

Cyclophosphamide (Filaxis, SA, Argentina) was diluted to a stock concentration of 20 mg/ml by the addition of sterile distilled water. The stock solution was diluted at a concentration of 0.083 mg/ml in the drinking water, in order to reach a daily dose of 25 mg/kg of body weight per animal.

Losartan (Parafarm, Argentina) was diluted to a stock concentration of 166 mg/ml. The stock solution was diluted at a concentration of 0.5 or 0.67 mg/ml in the drinking water, so it can be reached a daily dose of 150 or 200 mg/kg of body weight per animal (M-406 and M-234p models, respectively).

The optimum doses of cyclophosphamide and losartan were established previously [11, 12, 44, 45].

### Tumors

M-234p and M-406 mouse triple negative mammary tumors established in our laboratory were used. M-234p is a type B moderately differentiated mammary adenocarcinoma [46] that shows a mixed pattern and develops lung metastasis. This tumor, which arose in a BALB/c female mouse, is maintained *in vivo* by serial s. c. passages in syngeneic mice, with an incidence of 100%. M-406 is a type B semi-differentiated mammary adenocarcinoma histologically similar to M-234p, spontaneously arisen in a CBi female mouse. It is maintained *in vivo* by serial i.p. passages in syngeneic mice, with an incidence of 100%.

### Experimental model

Adult BALB/c or CBi female mice were implanted orthotopically in the mammary fat pad with M-234p or M-406 tumor fragments (1-mm^3^), respectively. Five (M-234p) or eight (M-406) days later, when the tumors were palpable, the animals were distributed in four groups (M-234p: *n* = 6–7/group; M-406: *n* = 5–6/group) and treated as follows: Control: regular drinking water without drug administration; Cy: 25 mg/kg bw/day of Cy in the drinking water; Los: 200 mg/kg bw/day (M-234p) and 150 mg/kg bw/day (M-406) in the drinking water; Cy+Los: Cy and Los treatments combined. The drinking water for each experimental group was replaced every other day. The animals were controlled daily and weighed three times per week. Tumor sizes were measured with Vernier calipers, and tumor volumes were calculated as follows: V = 0.4 (ab^2^), where V = volume (mm^3^), a = largest diameter (mm), b = smallest diameter (mm). Different endpoints were set for survival analysis and to obtain tumor and blood samples. For survival analysis, each mouse was euthanized when it reached the maximum tumor volume ethically permitted; for tumor and blood samples collection, all the animals were euthanized when Control group tumors reached the maximum volume ethically permitted (day 22 and day 31 for M-406 and M-234p, respectively). On an additional experiment developed with the M-234p model, tumor samples of Cy and Cy+Los groups, were taken on day 42, a time point when mice from Control and Los groups had already been euthanized because of their tumor burden. In all the experiments, tumors were excised immediately after euthanasia, and processed for histology and immunohistochemistry.

### Histological and immunohistochemical studies

Excised tumors were fixed in 10% buffered formalin and were paraffin-embedded. Five- to six-micrometer thickness sections were obtained, deparaffinized and used for: 1) *Hematoxylin-Eosin staining*, using standard techniques, to analyze the structure and morphology of tumor capillaries; 2) *Immunohistochemical analysis*, antigens were unmasked by heating the sections at 95°C in 10 mmol/L citrate buffer with pH 6.0, for 15 min, and immunostaining was undertaken using the specific primary antibodies, CD4 (sc-13573, 1/400, Santa Cruz Biotechnology), CD8 (sc-18913, 1/400, Santa Cruz Biotechnology), Foxp3 (#14477480, 1/50, eBioscience), Ki67 (#12202, 1/400, Cell Signaling), HIF-1α (MA1-16518, 1/5000, Thermo Fisher) and αSMA (ab5694, 1/300, Abcam). The antigen was visualized using the Vectastain Elite ABC kit (Vector Lab., Burlingame, CA), following manufacturer’s instructions. Sections were then treated with 3,3′-diaminobenzidine (Sigma) as a chromogen, for 5 min, and were lightly counterstained with soft hematoxylin. Negative controls were performed avoiding the primary antibody. Slides were washed in tap water, dehydrated, and mounted with glass coverslips; 3) *Apoptosis*, tumor sections were immunostained by the TUNEL method (*In Situ* Cell Death Detection Kit, Roche); 4) *Collagen quantification*, tumor sections were stained with Picro-Sirius Red staining (Direct Red 80 Sigma).

#### Quantification

The positive cells for CD4, CD8, Foxp3, Ki67 and HIF-1α were counted in 30 fields (1000×); αSMA and collagen areas were evaluated with Image J software in 30 fields (1000×).

### Flow cytometry

Blood samples and tumor cells suspensions were used to evaluate circulating and tumor infiltrating CD4 (#550954, 1/400, BD Bioscience), CD8 (#22150084, 1/200, Immunotools), Treg (CD4^+^, Foxp3^+^ [#560401, 1/300, BD Bioscience]) and Th17 (CD4^+^, IL-17^+^ [#559502, 1/400, BD Bioscience]) positive cells by flow cytometry using a FACSARIA II cytometer. Acquired data were analyzed with flowing software version 2.

### Statistics

Data obtained was analyzed using ANOVA and Tukey-Kramer Multiple Comparison tests, Kruskal-Wallis and Dunn’s post-test, and Log-rank tests were used to examine the differences between groups with GraphPad Prism version 3.0 (GraphPad Software, San Diego, CA).

## SUPPLEMENTARY MATERIALS


