# Supplementary material for: Losartan improves the therapeutic effect of metronomic cyclophosphamide in triple negative mammary cancer models
Source: Oncotarget. 2020 Aug 11;11(32):3048–60. doi: 10.18632/oncotarget.27694 (PMC7429183; doi:10.18632/oncotarget.27694)
Supplement: Supplementary file 1 [file oncotarget-11-3048-s001.pdf]

Losartan improves the therapeutic effect of metronomic cyclophosphamide in triple negative mammary cancer models

SUPPLEMENTARY MATERIALS

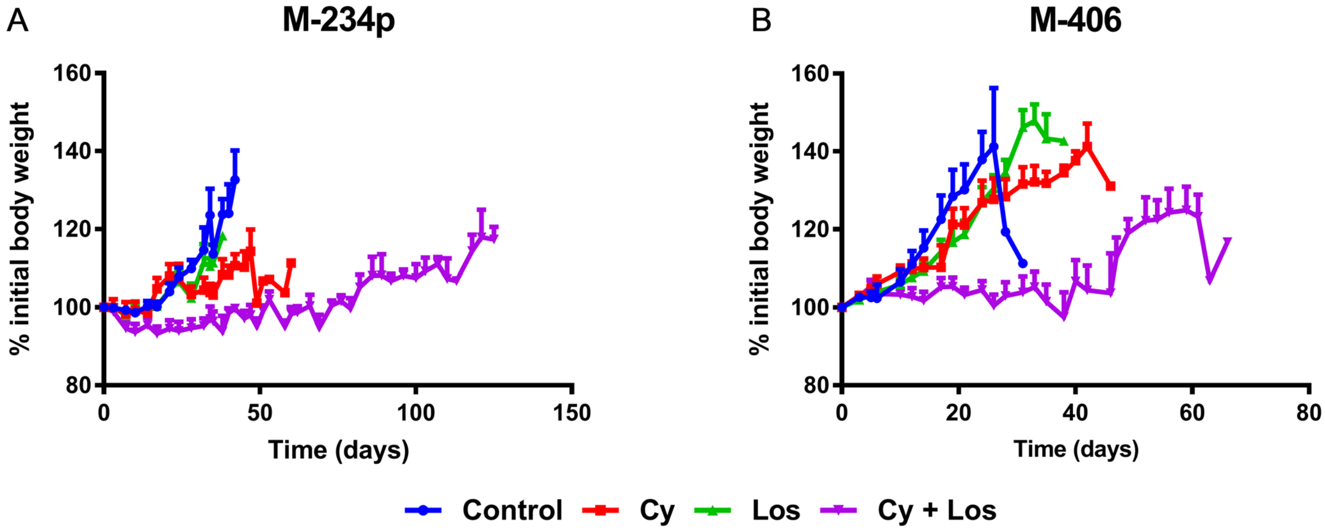

Supplementary Figure 1: Evolution of body weight: data for each time point are mean ± SEM of the percentage of initial body weight. (**A**) M-234p, (**B**) M-406.
